# shRNA-interfered of Nrf2 reveals a critical role for Keap1-Nrf2 signaling pathway during effects of zearalenone induced oxidative stress in IPEC-J2 cells

**DOI:** 10.5713/ab.24.0368

**Published:** 2024-08-26

**Authors:** Qun Cheng, Shu Zhen Jiang, Li Bo Huang, Wei Ren Yang

**Affiliations:** 1 Department of Animal Sciences and Technology, Qingdao Agricultural University, Qingdao Shandong 266109, China; 2 Shandong Provincial Key Laboratory of Animal Biotechnology and Disease Control and Prevention, Department of Animal Sciences and Technology, Shandong Agricultural University, Taian, Shandong 271018, China

**Keywords:** IPEC-J2, Keap1-Nrf2 Signaling Pathway, Nrf2-shRNA, Oxidative Stress, Zearalenone

## Abstract

**Objective:**

This study aims to verify the protective effect of the Kelch-like ECH-associated protein1 (Keap1)–nuclear factor erythroid 2–related factor 2 (Nrf2) signaling pathways by studying the effect of plasmids containing Nrf2–small hairpin RNA (shRNA) interference down-regulation of Nrf2 on zearalenone (ZEA)–induced intestinal porcine epithelial cells (IPEC-J2) oxidative stress.

**Methods:**

We constructed an IPEC-J2 model that interferes with Nrf2 expression, set blank (control), negative control group (Sh-control), positive control group (Sh-Nrf2), and added 10, 20, and 40 μmol/L ZEA experimental group (Sh-Nrf2+ZEA10, Sh-Nrf2+ZEA20, and Sh-Nrf2+ZEA40).

**Results:**

The study results showed that, compared with the Sh-Nrf2 group, ZEA significantly increased the apoptosis rate of IPEC-J2 in a time- and dose-dependent manner. Compared with the Sh-Nrf2 group, the activities of total superoxide dismutase and glutathione peroxidase and relative expressions of Keap1 at mRNA and protein level in the Sh-Nrf2+ZEA20 and Sh-Nrf2+ZEA40 groups were significantly reduced, the malondialdehyde level, and the fluorescence intensity around and within the nucleus of reactive oxygen species and Nrf2, and the relative expressions of Nrf2, quinone oxidoreductase 1, and hemeoxygenase 1 at mRNA and protein level significantly increased.

**Conclusion:**

These results further prove that interfering with the expression of Nrf2 in IPEC-J2 cells affected the activation of the Keap1-Nrf2 signaling pathway and reduced the ability of cells to resist ZEA-induced oxidative stress. Therefore, the Keap1-Nrf2 signaling pathway had an important protective effect in ZEA-induced intestinal oxidative stress.

## INTRODUCTION

Zearalenone (ZEA) is a macrocyclic β-resorcylic acid lactone, and a non-steroidal estrogen fungal toxin isolated for the first time [1,2]. ZEA is synthesized by several fungi, such as Fusarium graminearum, Fusarium culmorum, Fusarium cerealis, Fusarium equiseti, and Fusarium semitectum [3]. Different degrees of ZEA pollution have been observed in cereals all over the world, which has brought huge economic losses to animal production and breeding and threatened human health [4]. It is widely known that ZEA has low acute toxicity. However, when animals are fed contaminated feed over a long period of time, it can lead to various types of toxicity, such as reproductive toxicity [5], genetic toxicity, and immunotoxicity. Among these, oxidative stress is one of the main pathways through which ZEA causes toxicity, both in laboratory experiments and in living organisms [6,7]. ZEA and its metabolites can increase reactive oxygen species (ROS) levels through the mitochondrial pathway, which may disrupt the cell’s own antioxidant defense system, leading to lipid, protein, and DNA oxidation, further leading to oxidative stress [8]. It has been reported that ZEA induces a decrease in antioxidant enzyme activity in the intestine of weaned sows [9]. Additionally, studies have discovered that ZEA decreases glutathione-S-transferase activity in the kidneys and testicles of mice. These findings suggest that oxidative stress is one of the harmful effects brought on by ZEA [10].

It is widely recognized that the intestinal epithelium functions as the primary physical barrier of the body. Upon consuming food tainted with ZEA, animals experience initial exposure of their small intestine to elevated levels of ZEA toxins, which will inevitably result in detrimental effects on the health of the small intestine [11,12]. An increasing number of studies have found that ZEA may impair the function of animal intestinal organs and tissues, seriously damaging animal health [9,13]. It has been demonstrated that ZEA can alter intestinal villi structure and decrease connexin gene expression, both of which compromise the integrity and function of intestinal epithelial cells [12]. However, there is relatively little research on the mechanism of ZEA induced oxidative stress in the small intestine of piglets. Since the interaction of intestinal oxidative stress and toxins with the intestinal mucosa can be studied using the intestinal porcine epithelial cell line IPEC-J2, IPEC-J2 was chosen as the research subject in this study.

A condition known as oxidative stress occurs when the body produces excessive amounts of highly active molecules, such as ROS, which can damage tissue over time and cause a number of diseases as a result of unfavorable stimulation and oxidation [14]. The Kelch-like ECH-associated protein1 (Keap1)- nuclear factor erythroid 2-related factor 2 (Nrf2) signaling pathway is crucial for the oxidative stress system and is essential for shielding cells from oxidative stress [15,16]. It is widely known that Nrf2 plays a significant role in preserving intracellular homeostasis by controlling the production of essential antioxidant enzymes, including hemeoxygenase 1 (Ho1) and quinone oxidoreductase 1 (Nqo1) [17]. Therefore, the Nrf2 antioxidant stress signal is a potential therapeutic target to prevent or treat oxidative stress-related diseases [18]. This study aims to use shRNA interference to inhibit the expression of the *Nrf2* gene in IPEC-J2, observe the effect of Nrf2 expression inhibition on ZEA induced oxidative stress in IPEC-J2, elucidate the mechanism of the Keap1-Nrf2 signaling pathway in the ZEA-induced oxidative stress model of IPEC-J2, and better understand how ZEA affects cell health.

## MATERIALS AND METHODS

### Preparation of intestinal porcine epithelial cells culture

The IPEC-J2 were purchased from Beina Biological Co., Ltd. (Xinyang, China), which were prepared from a single batch and stored in sterilized tubes in liquid nitrogen. Upon each assay, the cell cryotube containing IPEC-J2 was removed from the liquid nitrogen and immediately put into the 37°C water bath for 1.5 min, and then the outside of the tube was sterilized by 70% ethanol before being placed on an ultraclean platform. The cell content in the tube was then transferred into a 1.5 mL centrifuge tube and centrifuged at 1,000 g for 5 min. After removal of the supernatant, the IPEC-J2 were cultured with 2 mL of Dulbecco’s modified eagle medium (DMEM) high glucose (11995-065; GIBCO Co., Ltd, Shanghai, China) containing 10% fetal bovine serum (FBS, 16000-044; GIBCO Co., Ltd, China) and 1% penicillin-streptomycin (P/S, 15140-163; GIBCO Co., Ltd, China). The incubation was conducted at 37°C in a cell incubator containing 5% CO_2_ before use in each assay as described below.

### Generation of IPEC-J2 stably expressing Nrf2-shRNA and ZEA treatment of IPEC-J2

IPEC-J2 were seeded in 6-well plates (2×10^5^ cells per well) and allowed to adhere for 24 h before transfection. Using jetPRIME transfection assay kit, (Polyplus, Illkirch, France), cells were transfected with plasmids containing Nrf2-small hairpin RNA (ShRNA) directed against pig Nrf2 and the target sequence was GATGACAG-TGAACTCAT TAAACTCGAGTTTAATGAGTTCACTGTCATC (Cyagen Biosciences, Guangzhou, China). The new plasmid was termed Sh-Nrf2. Non-targeting vector-control shRNA and the sequence was CCTAAGGTTAAGTCGCCCTCGCTC GAGCGAGGGCGACTTAAC-CTTAGG (Cyagen Biosciences, China) and was termed Sh-control. Transfection was continued for 24 h, followed by a 24-h recovery in the complete medium. At 48 h post-infection, puromycin (6.5 g/mL) was added to the culture medium for selection and further characterization. At 24 h post-infection, the transfection efficiency of IPEC-J2 expressing Nrf2-shRNA was assessed by gene and protein expression detection. The stable cell lines with reduced Nrf2 expression were used in cell biology experiments [19].

For each assay conducted below, the above prepared IPEC- J2 cells expressing Nrf2-shRNA were first distributed into 6 well plates (1×10^6^ cells per well), to which aliquots of ZEA solution [20] were added so that the concentrations of ZEA in the culture reached 0 (Sh-Nrf2), 10 (Sh-Nrf2+ZEA10), 20 (Sh-Nrf2+ZEA20), and 40 (Sh-Nrf2+ZEA40) μmol/L, respectively. The blank (Control) and non-targeting vector-control shRNA (Sh-control) were set at the same time. After mixing, the cultures were incubated at 37°C for 24 h, then the cells were harvested and processed further according to the descriptions in each assay. Each treatment was repeated at least three times in the experiment.

### Determination of the effects of ZEA on cell viability of IPEC-J2

The cell suspension was inoculated in a 96 well plate with a cell concentration of 1×10^5^/mL, 100 μL per well, and 3 replicates in each group. After the cells were stabilized for 24 h, the original medium was discarded, and then the cells were treated with different concentrations of ZEA, and 10 μL cell counting kit (CCK-8) solution was added into each well at 24 h, respectively. The cells were further incubated in a 5% CO_2_ cell incubator at 37°C for 1.5 hours. The absorbance at 450 nm was detected by an enzyme-linked immunosorbent assay.

### Determination of the effects of ZEA on cell apoptosis of IPEC-J2

A flow cytometry assay was conducted to determine the effects of ZEA on cell apoptosis in IPEC-J2. At the end of the incubation with varying concentrations of ZEA as described above, the IPEC-J2 were collected using 0.25% trypsin, centrifuged to remove trypsin, washed with 2 mL cold phosphate-buffered saline (PBS) twice, and resuspended into 100 μL 1×binding buffer. The harvested IPEC-J2 were then stained with PI (C1052-2; Beyotime, Shanghai, China) and FITC Annexin V (C1052-3; Beyotime, China) at 25°C in the dark for 15 min. After the reaction, 400 μL of 1×binding buffer was added to each tube and mixed well. The samples are subsequently analyzed using a BD FACSCalibur flow cytometer (FACSCalibur, BD, San Jose, CA, USA) with Flowjo 7.6 software.

### Determination of antioxidant enzyme activity

At the end of 24 h ZEA treatment as described above, IPEC-J2 were obtained (the same as above). The samples were collected to analyze the total superoxide dismutase (T-SOD) and glutathione peroxidase (GSH-PX) activities and malondialdehyde (MDA) concentration using the methods described by Jiang et al [21] with T-SOD A001-1, GSH-PX A005, and MDA A003 Assay Kits, respectively (Nanjing Aoqing Biotechnology Co., Ltd, Nanjing, China).

### Determinations of the effects of ZEA on Keap1-Nrf2 signal pathway and on relative mRNA expressions of related genes

Total RNA was extracted from the cells using RNAiso Plus (Applied TaKaRa, Dalian, China) according to the manufacturer’s instructions. The purity and concentration of the RNA were determined using an Eppendorf Biophotometer (DS-11; Denovix, Wilmington, DE, USA) at an absorbance ratio of 260/208 nm. Total RNA was reversely transcribed to cDNA using a Reverse Transcription System Kit (PrimeScript RT Master Mix, RR036A; Applied TaKaRa, China), and the resultant cDNA was divided into two subsamples. cDNA was stored at −20°C until it was used for transcriptome sequencing and real-time quantitative polymerase chain reaction (qRT-PCR) quantification.

One cDNA subsample was sequenced at Novogene Co. (Beijing, China) with the Illumina Hiseq 2000 sequencing system (San Diego, CA, USA) and the data obtained was aligned to the reference genome by HISAT2 software, with the image data being converted into reads by CASAVA base recognition. Based on the positional information of the gene on the reference genome, the number of reads covered by each entire gene was counted, which was then used to quantify the amount of gene expression using the feature Counts of the subread software.

The other subsample of cDNA was used for qRT-PCR quantification. The reaction mixture contained 10 μL of SYBR, 0.4 μL of Dye II, 1.9 μL of forward and reverse primers, 6 μL of nuclease-free water and 2 μL of cDNA. All the primers were designed by Sangon Biological Engineering Technology and Services Co. Ltd. (Shanghai, China) and synthesized at the Beijing Genomics Institute (BGI, Beijing, China). The optimized qRT-PCR protocol included an initial denaturation step at 95°C for 30 s, followed by 43 cycles at 95°C for 5 s, 60°C for 34 s, 95°C for 15 s, 60°C for 60 s, and 95°C for 15 s. The qRT-PCRs were conducted on an AB 7500 Real Time PCR System (Applied Biosystems, Foster City, CA, USA). The relative expression level of Keap1, Nrf2, Nqo1, Ho1, and β-actin mRNA was calculated using the 2^−ΔΔCt^ method [22]. The analysis was repeated three times per sample. The sequence and production length of primers are presented in Table 1.

### Determination of the effect of ZEA on ROS and Nrf2 distribution in IPEC-J2

The IPEC-J2 were first seeded on microslides, which were then subjected to ZEA treatment as described above and subsequently fixed with 4% paraformaldehyde for 1 h and permeated with Triton X-100 (0.5%) for 10 min at room temperature. The resultant cells were processed in the following steps: washing with PBS thrice for 5 min each, blocking with 10% FBS for 1 h, incubating with Nrf2 (1:500, ab89443; Abcam, Cambridge, UK), ROS (1:200, ab236409; Abcam, UK) at 4°C overnight, washing with PBS three times, mixing with diluted Alexa Fluor 555-labeled goat anti-rabbit immunoglobulin G (IgG) (1:200, ab150079; Abcam, UK) at 37°C in the dark for 1 h, and washing with PBS. The above processed cells were subsequently added to appropriate Hoechst 33342 (C1022; Beyotime, China), mixed for 5 min, and then washed with PBS. The processed samples were examined under a confocal microscope (FLUOVIEW FV3000; Olympus, Tokyo, Japan).

### Determination of the effects of ZEA on protein expression of the Nrf2, Keap1, Nqo1, and Ho1

After 24 h incubation with ZEA, the culture was added to 500 μL of 0.25% trypsin and centrifuged at 1,200 g for 5 min. The resultant pellet was rinsed with 2 mL of PBS and centrifuged again (1,200 g, 5 min), which was subsequently extracted with lysate containing PMSF (1 mmol/L; Beyotime, China) using the manufacturer’s protocol. The extract was determined for total protein content first using a bicinchoninic acid protein assay kit (Beyotime, China) and then subjected to Western blotting to determine the protein expression of relevant mRNA as described below. Each sample with 60 μg of protein was loaded on polyacrylamide gels and subjected to electrophoresis for 1.5 h. The separated bands were then transferred to immobilonp transfer membranes (Solarbio, Beijing, China) that were first blocked in 10% skimmed milk powder for 2 h, washed with Tris Buffered Saline Tween (TBST, pH 7.6) three times, and then incubated with monoclonal mouse antibody β-actin (1:1,500, SC-47778; Santa Cruz, CA, USA), polyclonal rabbit antibody Nrf2 (1:1,000, ab92946; Abcam, UK), polyclonal rabbit antibody Keap1 (1:1,000, ab196346; Abcam, UK), Nqo1 (1:500, ab2346; Abcam, UK), and Ho1 (1:500, ab13248; Abcam, UK) overnight at 4°C. After incubation, the membrane was washed with TBST three times for 5 min each and further incubated with goat anti-rabbit IgG (1:5,000, Thermo Pierce 31210; Thermo Fisher Scientific, USA) and goat anti-mouse IgG (1:5,000, Thermo Pierce 31160; Thermo Fisher Scientific, USA) in diluted secondary antibody dilution buffer (Beyotime, China) at room temperature for 2 h. Following washing with TBST for 30 min, the membranes were immersed in a high-sensitivity luminescence reagent (BeyoECL Plus; Beyotime Biotechnology, China), exposed to film using a Fusion FX imaging system, and analyzed using FusionCapt Advance FX7 software (Beijing Oriental Science and Technology Development Co., Ltd., Beijing, China). The concentration of proteins was determined using Image-Pro Plus 6.0 (Media Cybernetics, Inc., Rockville, MD, USA).

### Statistical analysis

All data was analyzed using the general linear model procedure of SAS 9.2 (SAS Institute Inc., Cary, NC, USA). An analysis of variance was performed by one-way analysis. The data were initially analyzed as a completely randomized design, with treatment as the fixed effect and individual piglets as a random factor. The significance of differences among treatments was tested using Duncan’s multiple range tests, and significance was declared at p<0.05.

## RESULTS

### Effects of ZEA on cell viability of the IPEC-J2

**Figure 1:**
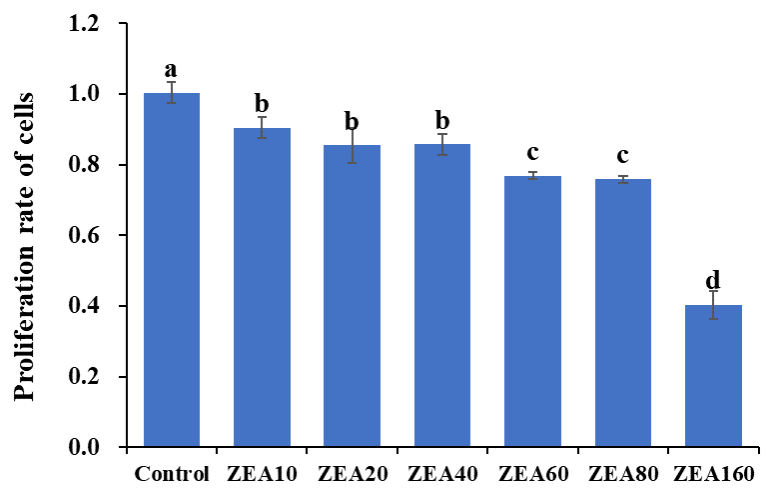
Effects of zearalenone on cell viability of the intestinal porcine epithelial cells (IPEC-J2) after 24 h. The IPEC-J2 exposed to ZEA at 0 (Control), 10 (ZEA10), 20 (ZEA20), 40 (ZEA40), 60 (ZEA60), 80 (ZEA80), and 160 (ZEA160) μmol/L for 24 h. ^a–d^ Value columns with different small letter superscripts mean significant difference (p<0.05).

After different concentrations of ZEA (0, 10, 20, 40, 80, and 160 μmol/L) were treated with IPEC-J2 for 24 h, the changes in cell viability were detected by the CCK-8 method. The results are shown in Figure 1. With the increase in ZEA concentration, cell viability decreased significantly (p<0.05). When the ZEA concentration was 160 μmol/L, the cell viability was the lowest.

### Effects of ZEA on apoptosis of the IPEC-J2

**Figure 2:**
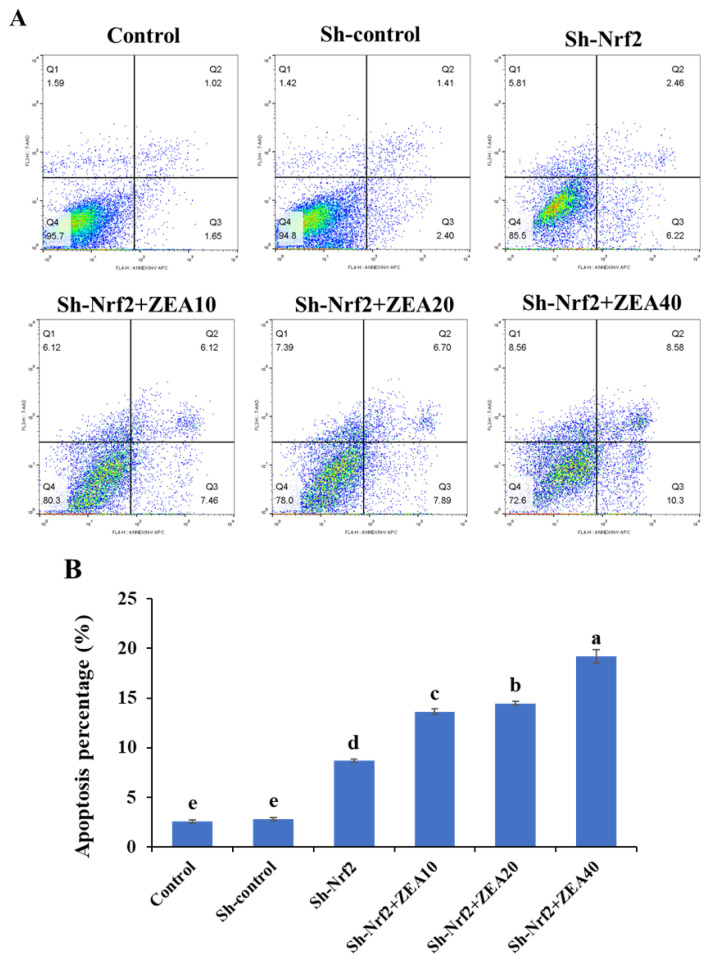
Effects of zearalenone on the apoptosis of intestinal porcine epithelial cells (IPEC-J2) after interfering with the nuclear factor erythroid 2-related factor 2 (Nrf2) expression. The experiment set blank (control), negative control group (Sh-control), positive control group (Sh-Nrf2). The interfering Nrf2 expression IPEC-J2 cells were treated with ZEA 10 μmol/L (Sh-Nrf2+ZEA10), 20 μmol/L (Sh-Nrf2+ZEA20), and 40 μmol/L (Sh-Nrf2+ZEA40) for 24 h, then collected to assess the apoptosis. ^a–e^ Bars with different letters difer (p<0.05).

Cell apoptosis analysis by flow cytometry showed that, there was no significant difference in apoptosis rate between the Sh-control group and Control group, and the apoptosis rate of the Sh-Nrf2 group was significantly higher than that of the Control group and the Sh-Control group (Figure 2). Compared with the Sh-Nrf2 group, with the increase in ZEA concentration, the apoptosis rate of IPEC-J2 that interfered with Nrf2 expression increased significantly (p<0.05) and showed a certain dose dependence. The apoptosis rate of the Sh-Nrf2+ZEA40 treatment group was the highest, which indicated that interfering Nrf2 expression and a high concentration of ZEA co-induced apoptosis.

### Effects of ZEA on antioxidant enzyme activity of the IPEC-J2

**Figure 3:**
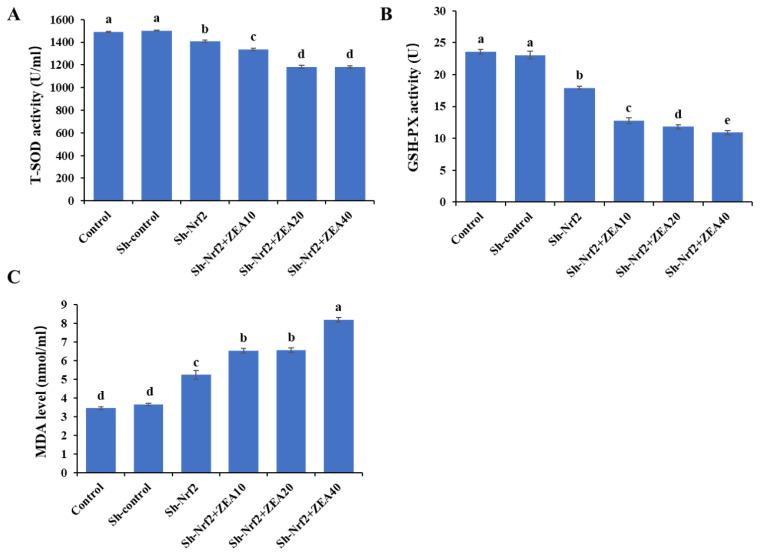
Effects of zearalenone on antioxidant capacity in intestinal porcine epithelial cells (IPEC-J2) after interfering with Nrf2 expression. The experiment set blank (Control), negative control group (Sh-control), positive control group (Sh-Nrf2). The interfering Nrf2 expression IPEC-J2 cells were treated with ZEA 10 μmol/L (Sh-Nrf2+ZEA10), 20 μmol/L (Sh-Nrf2+ZEA20), and 40 μmol/L (Sh-Nrf2+ZEA40) for 24 h, then collected to assess the antioxidant capacity levels of the total superoxide dismutase (T-SOD) (A), glutathione peroxidase (GSH-Px) (B), and malondialdehyde (MDA) (C). ^a–e^ Bars with different letters difer (p<0.05).

The antioxidant enzyme activity results showed that, compared with the control group, the T-SOD and GSH-PX activities and MDA levels of IPEC-J2 cells in the Sh-control group were not significantly different (Figure 3). Compared with the Control and Sh-control groups, the Sh-Nrf2 group interfered with the expression of the *Nrf2* gene, significantly reduced (p<0.05) the T-SOD and GSH-PX activities of IPEC-J2 (Figure 3A and 3B), and significantly increased (p<0.05) the MDA level (Figure 3C). ZEA has a significant inhibitory effect on the antioxidant enzyme activity of IPEC-J2 in the Sh-Nrf2 group. Compared with the Sh-Nrf2 group, the T-SOD and GSH-PX activities of IPEC-J2 cells in the ZEA-treated group were significantly decreased (p<0.05), and the MDA level was significantly increased (p<0.05). In the ZEA treatment group, the activities of T-SOD and GSH-PX of IPEC-J2 cells in the Sh-Nrf2+ZEA40 group were the lowest, and the level of MDA was the highest (p<0.05). Interfering with *Nrf2* gene expression in cells reduced the activity of cellular antioxidant enzymes, and the addition of ZEA further reduced the activity of cellular antioxidant enzymes. The ability of cells interfering with *Nrf2* gene expression to resist ZEA toxicity was reduced.

### Effects of ZEA on the relative expression of Keap1, Nrf2, Nqo1, Ho1 mRNA

**Figure 4:**
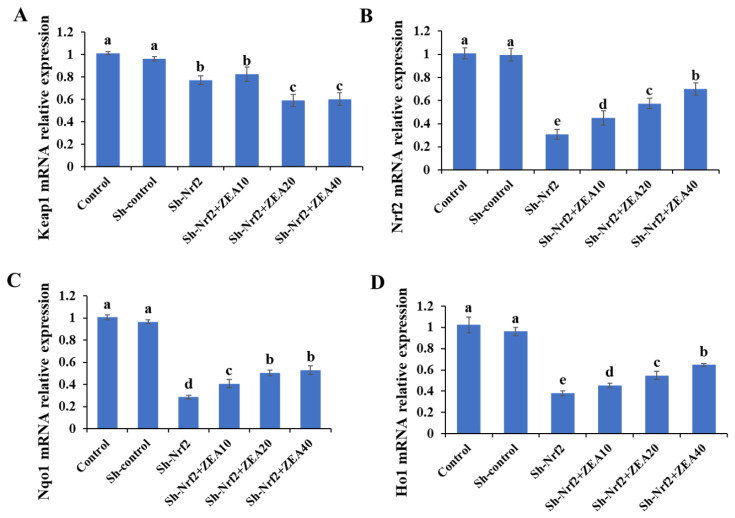
Effects of zearalenone on the relative mRNA expression of IPEC-J2 cells after interfering with Nrf2 expression. The experiment set blank (Control), negative control group (Sh-control), positive control group (Sh-Nrf2). The interfering Nrf2 expression IPEC-J2 cells were treated with ZEA 10 μmol/L (Sh-Nrf2+ZEA10), 20 μmol/L (Sh-Nrf2+ZEA20), and 40 μmol/L (Sh-Nrf2+ZEA40) for 24 h, then collected to assess the mRNA levels of the Kelch-like erythroid cell-derived protein with CNC homology-associated protein 1 (Keap1) (A), Nrf2 (B), quinone oxidoreductase 1 (Nqo1) (C), and hemeoxygenase 1 (Ho1) (D). ^a–e^ Bars with different letters difer (p<0.05).

RNA-seq results indicate that, compared with the control group, the relative expressions of Keap1, Nrf2, Nqo1, and Ho1 mRNA in IPEC-J2 cells in the Sh-control group were not significantly different (Figure 4). Compared with the Control and Sh-control groups, the Sh-Nrf2 group significantly reduced the relative expressions of Keap1, Nrf2, Nqo1, and Ho1 mRNA in IPEC-J2 cells after interfering with the expression of the *Nrf2* gene (p<0.05). Compared with the Sh-Nrf2 group, the relative mRNA expression of Keap1 in the Sh-Nrf2+ZEA20 and Sh-Nrf2+ZEA40 treatment groups was significantly reduced (p<0.05; Figure 4A), while the relative mRNA expression of Nrf2, Nqo1, and Ho1 was significantly increased (p<0.05; Figure 4B, 4C, and 4D). However, the relative expressions of Nrf2, Nqo1, and Ho1 mRNA in IPEC-J2 in the ZEA-treated group were always lower than those in the control and Sh-control groups.

### Effects of ZEA on the relative expression of Keap1, Nrf2, Nqo1, Ho1 Proteins

**Figure 5:**
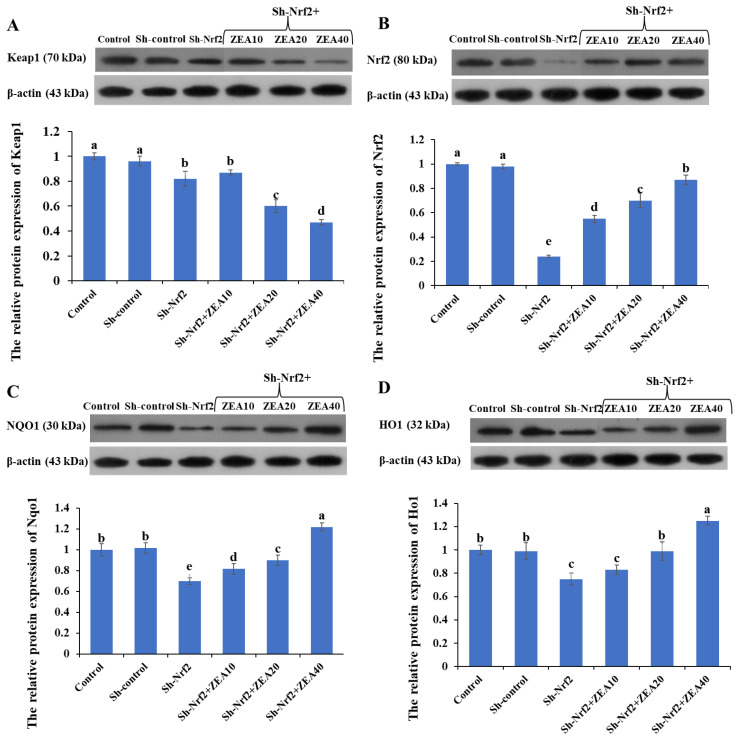
Effects of zearalenone on the relative protein expression of IPEC-J2 cells after interfering with Nrf2 expression. The experiment set blank (Control), negative control group (Sh-control), positive control group (Sh-Nrf2). The interfering Nrf2 expression IPEC-J2 cells were treated with ZEA 10 μmol/L (Sh-Nrf2+ZEA10), 20 μmol/L (Sh-Nrf2+ZEA20), and 40 μmol/L (Sh-Nrf2+ZEA40) for 24 h, then collected to assess the protein levels of Keap1 (A), Nrf2 (B), Nqo1 (C), and Ho1 (D) using western-blot analysis. ^a–e^ Bars with different letters difer (p<0.05).

Western blot analysis revealed positive bands of appropriate sizes for all the studied genes (*Keap1*, *Nrf2*, *Nqo1*, *Ho1*, and *β-actin*; Figure 5). Compared with the control group, the relative protein expression of Keap1, Nrf2, Nqo1, and Ho1 in IPEC-J2 in the Sh-control group was not significantly different. Compared with the control group and the Sh-control group, the relative protein expression of Keap1, Nrf2, Nqo1, and Ho1 in the Sh-Nrf2 group was significantly decreased (p<0.05). Compared with the Sh-Nrf2 group, the relative protein expression of Keap1 was significantly decreased (p<0.05), the relative protein expression of Nrf2 and Ho1 was significantly increased (p<0.05) in the Sh-Nrf2+ZEA20 and Sh-Nrf2+ ZEA40 treatment groups. Compared with the Sh-Nrf2 group, the relative protein expression of Nqo1 in the ZEA treatment group increased significantly (p<0.05).

### Localizations of reactive oxygen species

**Figure 6:**
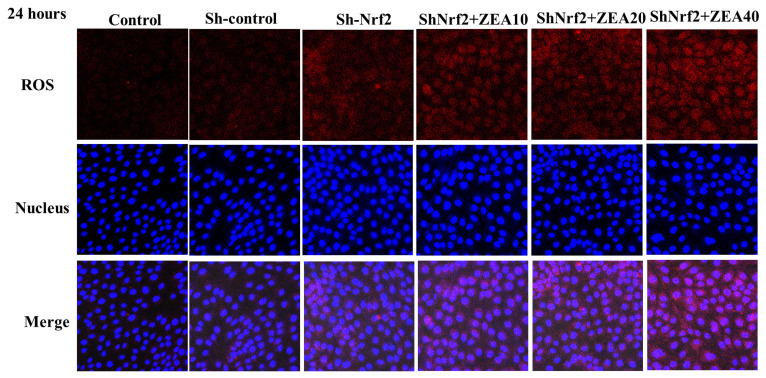
Effects of zearalenone on the expression of reactive oxygen species (ROS) in IPEC-J2 cells after interfering with nuclear factor erythroid 2–related factor 2 (Nrf2) expression for 24 h. The experiment set blank (Control), negative control group (Sh-control), positive control group (Sh-Nrf2). Immunostaining of ROS of the interfering Nrf2 expression IPEC-J2 cells were treated with ZEA 10 μmol/L (Sh-Nrf2+ZEA10), 20 μmol/L (Sh-Nrf2+ZEA20), and 40 μmol/L (Sh-Nrf2+ZEA40) for 24 h and stained by indirect immunofluorescence and observed under a light microscope (40×).

The results of intracellular ROS immunofluorescence localization are shown in Figure 6. ROS in the Control and Sh-control groups were expressed in small amounts, mainly in the cytoplasm and a small amount in the nucleus. Compared with the Control and Sh-control groups, the ROS immunofluorescence intensity of the Sh-Nrf2 group was significantly enhanced, and the ROS expression increased significantly. Compared with the Sh-Nrf2 group, with the increase in ZEA concentration, the fluorescence intensity around and in the nucleus of the ZEA treatment group increased significantly, indicating that the expression of ROS increased significantly. When cells were treated with 40 μmol/L ZEA, the expression of intracellular ROS reached its highest level.

### Localizations of Nrf2

**Figure 7:**
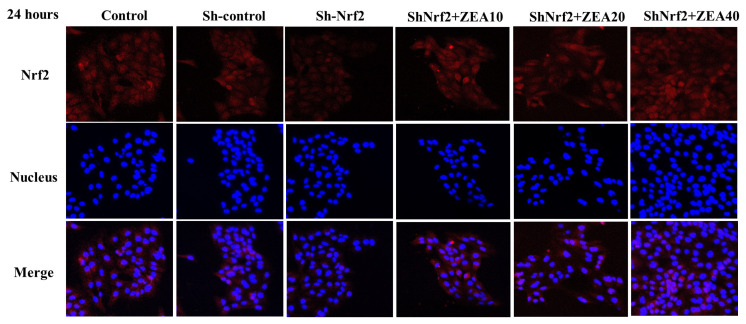
Effects of zearalenone on the expression of nuclear factor erythroid 2–related factor 2 (Nrf2) in IPEC-J2 cells after interfering with Nrf2 expression for 24 h. The experiment set blank (Control), negative control group (Sh-control), positive control group (Sh-Nrf2). Immunostaining of Nrf2 of the interfering Nrf2 expression IPEC-J2 cells were treated with ZEA 10 μmol/L (Sh-Nrf2+ZEA10), 20 μmol/L (Sh-Nrf2+ZEA20), and 40 μmol/L (Sh-Nrf2+ZEA40) for 24 h and stained by indirect immunofluorescence and observed under a light microscope (40×).

The results of immunofluorescence localization of the key gene *Nrf2* in the Keap1-Nrf2 classical signaling pathway in cells are shown in Figure 7. Nrf2 was mainly distributed in the cytoplasm and a small amount in the nucleus in the control and Sh-control groups. Compared with the control and Sh-control groups, the fluorescence intensity around the nucleus of the Sh-Nrf2 group was significantly reduced, indicating that the expression of Nrf2 was significantly reduced. Compared with the Sh-Nrf2 group, with the increase in ZEA concentration, the fluorescence intensity around and in the nucleus of the ZEA treatment group increased significantly, indicating that the expression of Nrf2 increased significantly.

## DISCUSSION

ZEA has a wide range of toxic effects, and exposure to ZEA can detrimentally affect multiple systems in the body. ZEA exposure promotes intracellular ROS production and cellular lipid peroxidation, leading to oxidative stress damage in cells. RNA interference (RNAi) is a widely documented phenomenon in nature in recent years that can silence specific genes. Small hairpin RNA (shRNA) mainly participates in RNAi and regulates gene expression in a specific manner. Previous research has shown that ZEA can activate the Nrf2 protein in the small intestinal tissue of weaned piglets to translocate it from the cytoplasm into the nucleus and activate the expression of downstream antioxidant and protective genes, thereby reducing ZEA-induced oxidative stress in cells [13]. In this study, IPEC-J2 cells and shRNA interference technology were used to inhibit Nrf2 expression. We observed changes in apoptosis, intracellular ROS levels, and antioxidant enzyme activity of cells after ZEA exposure and further investigated the mechanism of Nrf2 in ZEA-induced oxidative stress toxicity in cells.

Our research revealed that ZEA (20 to 160 μmol/L) affected the morphology of IPEC-J2 cells, decreased their viability, and increased cell mortality, and these effects amplified as ZEA concentrations increased. Numerous studies have demonstrated that ZEA can stop cell cycle advancement, and restrict cell growth [20,23]. Therefore, monitoring the number of live cells in the presence of different ZEA concentrations was crucial for analyzing the inhibitory effects of ZEA on cell proliferation [24]. A CCK-8 was used to determine cell viability at different ZEA concentrations. A significant decrease in cell viability was observed with increasing ZEA concentrations. These results demonstrate that ZEA can have a significant impact on the activity of porcine small intestinal epithelial cells and provide a rationale for the ZEA concentration used in this study. To better understand the mechanism underlying ZEA toxin-induced IPEC-J2 poisoning, we selected ZEA doses of 10, 20, and 40 mol/L and an exposure duration of 24 h.

After 24 h of ShRNA administration to interfere with Nrf2 expression, IPEC-J2 cells showed a considerable increase in apoptosis rate. Similarly, studies have shown that downregulation of Nrf2 suppresses cell proliferation *in vitro* and tumor growth in mouse xenograft models [25]. Inhibiting the expression of Nrf2 and its downstream genes using RNAi can inhibit tumor growth [19]. In addition, downregulation of Nrf2 using antisense RNA can improve the sensitivity of cells to apoptosis [26]. Consistent with these findings, our study also revealed that the apoptosis rate of cells with altered Nrf2 expression treated with ZEA for 24 h was significantly higher than that of cells in the ShRNA interference Nrf2 expression group. Furthermore, apoptosis is enhanced in CaSki cells induced by chemical drugs and in which the endogenous *Nrf2* gene is silenced [19]. Similar to our findings, several previous studies have shown that mice lacking Nrf2 transcription factors are more susceptible to the harmful effects of foreign compounds and oxidants than wild-type mice [27]. Notably, shRNA interference can significantly disrupt *Nrf2* gene expression, suppress protein activity *in vitro*, and increase the sensitivity of IPEC-J2 cells to ZEA toxicity. Based on these observations, we speculate that the Nrf2-dependent Keap1-Nrf2 signaling pathway may be fully activated and therefore potentially participates in ZEA-induced apoptosis of IPEC-J2 cells.

Under normal conditions, the production and elimination of ROS are balanced. Excess levels of ROS result in lipid peroxidation and a number of disorders that affect the body’s health [28]. Previous studies have observed a 25-fold increase in ROS levels in A549-Nrf2 shRNA cells. Therefore, the primary consequences of shRNA-mediated silencing of Nrf2 are an increase in oxidative stress and an overall decrease in exogenous metabolic enzyme gene expression [29]. Numerous recently published studies have also shown that ZEA and its metabolites can increase ROS levels in the mitochondria and endoplasmic reticulum, thereby interfering with cellular antioxidant processes, producing oxidative stress in cells, and triggering cell apoptosis [30,31]. Immunofluorescence results of *Nrf2* gene-interfering IPEC-J2 cells treated with ZEA showed that ZEA significantly increased the expression of ROS in these cells. Similar studies have shown that suppressing Nrf2 expression can marginally increase the generation of intracellular ROS in GH3 cells. Additionally, when T-2 toxin was used to stimulate cells, silencing Nrf2 resulted in a further significant increase in ROS content and an increase in the susceptibility of the cells to the T-2 toxin [32,33]. In this study, we also discovered that the activity levels of the antioxidant enzymes T-SOD and GSH-PX in IPEC-J2 were remarkably decreased, and the MDA levels were significantly increased when shRNA interfered with *Nrf2* gene expression. When IPEC-J2 cells with downregulated *Nrf2* gene expression were treated with ZEA, the activities of the antioxidant enzymes T-SOD and GSH-Px decreased, while the MDA content increased further. This indicates that compared to normal cells, cells with reduced Nrf2 and antioxidant enzyme expression have a weaker ability to resist oxidative stress, are more affected by the toxicity of ZEA, and have a higher probability of cell death. Moreover, knockdown of the *Nrf2* gene [34] or cell-specific overexpression of Nrf2 can significantly inhibit the expression of glutathione and antioxidant genes, thereby affecting the response of cells to oxidative stress [19,29,35]. Therefore, we believe that Nrf2 may play a key role in controlling the changes in ROS levels induced by ZEA. ZEA causes oxidative stress by increasing ROS levels and decreasing antioxidant enzyme activity. The ability of cells to interfere with *Nrf2* gene expression and to resist ZEA toxicity decreased following ZEA administration. Further, ROS levels increased significantly, and the ability of cells to eliminate ROS free radicals decreased, eventually resulting in cell death.

The current study clearly demonstrates that Nrf2 is crucial for the control of Ho1 and Nqo1, two additional antioxidant enzymes. Specifically, our results show that after inhibiting Nrf2 expression in IPEC-J2 cells, the relative mRNA and protein expression levels of Nrf2, Ho1, and Nqo1 were much lower than those in control cells. This outcome indicates that blocking *Nrf2* gene expression suppresses the expression of antioxidant genes and lowers cellular antioxidant capability. Similarly, previous studies have found that Nqo1 almost loses its effect in *Nrf2* gene knockout mice [36], and Nrf2-deficient mice are more vulnerable to the toxicity of many compounds. In addition, the composition and induction levels of phase II detoxifying enzymes and endogenous enzymes, such as GST and Nqo1, were considerably diminished in *Nrf2* gene knockout mice [37]. ShRNA decreased Nrf2 expression levels in A549 cells and downregulated the production of Ho1 and Nqo1 [29]. Previous studies have found that in CaSki cells transfected with Nrf2 shRNA, the expression of Nrf2/ARE-dependent detoxification enzymes and glutathione-related enzymes, such as Ho1 and Nqo1, decreased significantly [19].

Under basal non-activated conditions, Nrf2 interacts with Keap1 to form a Keap1-Nrf2 complex, which limits *Nrf2*-mediated gene expression. Under oxidative stress, the phosphorylation of a variety of protein kinases enables Nrf2 to uncouple from Keapl and transfer to the nucleus. Nrf2 can then bind to the ARE regions of genes encoding phase II enzymes and antioxidative proteins, which in turn activates the cellular antioxidant defensive system [38]. When ZEA was used to treat cells that interfered with the expression of Nrf2, we found that the relative mRNA and protein expression levels of Nrf2, Ho1, and Nqo1 increased significantly, whereas the relative mRNA and protein expression levels of Keap1 were significantly reduced. Immunofluorescence results also showed that ZEA induced a significant increase in the expression of Nrf2, and Nrf2 expression reached its highest level in the Sh-Nrf2+ZEA40 group. These results indicate that ZEA activates the *Nrf2* gene and downstream target genes *Ho1* and *Nqo1* and inhibits the expression of Keap1. The Keap1-Nrf2 signaling pathway was clearly activated in cells and may play a role in resisting ZEA toxicity. Notably, the expression levels of Nrf2, Keap1, Ho1, and Nqo1 were significantly lower in the ZEA treatment group than in the control group. This result indicates that interfering with the expression levels of the *Nrf2* gene can significantly inhibit the activation of the Keap1-Nrf2 signaling pathway, thereby activating the protective effects of this signaling pathway on cells. We speculate that the activation of the Keap1-Nrf2 signaling pathway can boost the antioxidant capacity of cells to withstand the oxidative stress toxicity induced by ZEA. As the concentration of ZEA increased, Nrf2 expression progressively declined, and the effect of the Keap1-Nrf2 signaling pathway may also progressively decrease. Recent studies have shown that upregulation of Ho1 and Nqo1 synergistically promote tumor cell survival and inhibits apoptosis by regulating cellular glutathione levels [39]. However, little information is available regarding how ZEA affects the interference or knockout of Nrf2 in cells. In this study, interfering with *Nrf2* gene expression resulted in a decrease in the overall cellular *Nrf2* gene expression level, which inhibited the activation of the Keap1-Nrf2 signaling pathway, thereby reducing cell antioxidant capacity, and increasing apoptosis induced by ZEA-induced oxidative stress. The Keap1-Nrf2 signaling pathway clearly plays a crucial role in both health and oxidative stress in animals. During oxidative stress in IPEC-J2 cells caused by ZEA, the defense system of the Keap1-Nrf2 signaling pathway was activated and employed to counteract the toxicity of ZEA.

In conclusion, the ShRNA transfection plasmid interfered with the expression of Nrf2 in IPEC-J2 cells and reduced the ability of the cells to resist ZEA-induced oxidative stress. However, ZEA activated the expression of key genes such as *Nrf2* in the cells. Activation of the Keap1-Nrf2 signaling pathway and its crucial protective role in ZEA-induced oxidative stress in IPEC-J2 cells were further demonstrated, opening new avenues for investigating the detrimental effects of ZEA.

## Figures and Tables

**Table 1:** Real-time polymerase chain reaction primers and conditions

Gene	Genbank accession	Primer sequences(5′to3′)^1)^	Annealing (°C)
*Keap1*	NM_001114671.1	F:GTGTGTGCTCCATGTCATGAAT R:CTCCCCAAAGTGCATGTAGATG	58
*Nrf2*	XM_005671981.3	F:GAGTTAGATAGTGCCCCTGGAA R:ACTGGAGCACTATTACCCTGAG	60
*Nqo1*	NM_001159613.1	F:AAAAGCACTGATCATACTGGCC R:TTCTGGAGATGACGGGATTGAA	58
*Ho1*	NM_001004027.1	F:AGGTCCTCAAGAAGATTGCTCA R:CATCTCCAGAGTGTTCATTCGG	58
*β-actin*	XM_003124280.5	F:AGATCACTCCCCCAATGACAG R:AGAGCAAGAGAGGCATCCTG	58

*Keap1*, Kelch-like ECH-associated protein1; *Nrf2*, nuclear factor erythroid 2-related factor 2; *Ho1*, hemeoxygenase 1; *Nqo1*, quinone oxidoreductase 1.

1) F, forward primer; R, reverse primer.
